# Incidence and Histological Patterns of Thyroid Cancer in Tuzla Canton, Bosnia and Herzegovina: A Five-Year Retrospective Study, 2020–2024

**DOI:** 10.7759/cureus.113487

**Published:** 2026-07-28

**Authors:** Dzana Nuhanovic, Belkisa Izic, Emina Bajric Custo, Muamera Garagic, Maida Sljivic Husejnovic, Selma Caluk, Sabina Cemalovic, Danijel Gajic

**Affiliations:** 1 Department of Nuclear Medicine, University Clinical Center Tuzla, Tuzla, BIH; 2 Department of Nuclear Medicine and Radiology, University Clinical Center Tuzla, Tuzla, BIH; 3 Department of Family Medicine, Health Center Lukavac, Lukavac, BIH; 4 Faculty of Pharmacy, University of Tuzla, Tuzla, BIH; 5 Department of Nuclear Medicine, Kardiocentar Sarajevo, Tuzla, BIH; 6 Department of Cardiology, Cantonal Hospital Bihac, Bihac, BIH; 7 Department of Family Medicine, Health Center Orašje, Orašje, BIH

**Keywords:** epidemiology, incidence, papillary carcinoma, prevalence, thyroid neoplasms

## Abstract

Background: Thyroid cancer (TC) is the most common endocrine malignancy and is generally associated with a favorable prognosis. Its incidence has increased worldwide, whereas epidemiological data from Bosnia and Herzegovina, particularly Tuzla Canton, remain limited.

Aim: The aim of this study is to determine the incidence, five-year period prevalence, histological distribution, and basic demographic characteristics of TC in Tuzla Canton between 2020 and 2024.

Study design: This is a retrospective descriptive observational study.

Methods: Records of 483 patients evaluated by the Council for Benign and Malignant Thyroid Diseases at the University Clinical Center Tuzla between January 1, 2020, and December 31, 2024, were retrospectively reviewed. Patients were included if they had histopathologically confirmed TC, were aged ≥15 years, and resided in Tuzla Canton. Incidence and five-year period prevalence were calculated using the latest available population estimates.

Results: Of the 483 evaluated patients, 142 met the inclusion criteria. The cohort included 108 women (76%) and 34 men (24%), corresponding to a female-to-male ratio of 3.18:1. The median age at diagnosis was 56 years. Papillary TC was the most common type (n=105; 74%), followed by follicular (n=18; 13%), medullary (n=15; 10%), and anaplastic (n=4; 3%). The age-standardized average annual incidence was 6.99 per 100,000 population, while the five-year period prevalence was 32.98 per 100,000. Incidence was higher among women than among men and increased during the study period. The highest age-specific incidence was observed among women aged ≥75 years and men aged 65-74 years. Three deaths occurred during follow-up, of which two were directly attributable to TC; both TC-related deaths occurred in patients with anaplastic carcinoma.

Conclusion: TC incidence in Tuzla Canton was lower than reported global and European estimates but increased during the five-year study period. Papillary carcinoma predominated, particularly among women. Longer-term population-based studies and the establishment of a national thyroid cancer registry are needed to improve epidemiological surveillance in Bosnia and Herzegovina.

## Introduction

Thyroid cancer (TC) is the most common endocrine neoplasm and accounts for 1% of all malignancies [1-3]. Some of the factors that influence the development of TC are exposure to ionizing radiation, family history of nodules or goiter, positive family history of TC, and female sex [4,5]. TC constitutes about 4.1% of all global cancer diagnoses and is typically characterized by a favorable prognosis, with an estimated five-year survival rate of 98.5% [6]. According to data available on SEER (Surveillance, Epidemiology and Results) for the period from 1975 to 2012, there were 13.5/100,000 newly diagnosed cases of TC per year [2]. In addition, global TC cases are expected to rise by 44.1% from approximately 234,000 cases in 2019 to 337,000 cases in 2030, with the age-standardized incidence rate (ASIR) per 100,000 increasing by 16.3%, highlighting a growing public health concern [7].

According to GLOBOCAN (Global Cancer Observatory) data from 2020, the ASIR was 10 per 100,000 in women and 3 per 100,000 in men, with a mortality rate of less than 1 per 100,000 population [8]. In 2022, an estimated 821,214 new TC cases and 47,507 TC-related deaths were recorded worldwide, with higher age-standardized incidence and mortality rates (age-standardized mortality rate (ASMR)) observed in women (ASIR: 13.60; ASMR: 0.53 per 100,000) compared to men (ASIR: 4.60; ASMR: 0.35 per 100,000) [6]. This pattern of increasing incidence with stable mortality strongly suggests the influence of overdiagnosis, especially in highly developed countries where ultrasound and cytological diagnostics are widely used. More recent publications show that the incidence has stagnated in North America and Western Europe in the last decade [9,10]. More than 70 years ago, pathologists reported that TC, especially the papillary form, was a common autopsy finding, despite never causing symptoms. Harach et al. reported one or more occult papillary thyroid carcinoma foci in 36 of 101 examined thyroid glands, corresponding to 36% of individuals without clinically recognized TC during life [11-15].

Epidemiological data from Southeastern Europe remain incomplete, largely because of differences in cancer registration systems and limited availability of stable regional or national registries. However, available data from Croatia, Slovenia, and Serbia indicate a gradual increase in the incidence of TC in recent years, with the highest proportion of papillary carcinoma and almost negligible mortality. Bosnia and Herzegovina remains a region without reported data on the epidemiology of TC. The only available data are based on GLOBOCAN estimates from 2022 [16].

This retrospective, descriptive, observational, single-center study primarily aimed to determine the crude and age-standardized incidence and five-year period prevalence of TC in Tuzla Canton, Bosnia and Herzegovina, between 2020 and 2024. Secondary objectives were to assess temporal trends, histological distribution, and age- and sex-specific incidence patterns.

## Materials and methods

Study design and setting

This retrospective observational study was conducted in Tuzla Canton, a region in northeastern Bosnia and Herzegovina within the Federation of Bosnia and Herzegovina. According to the Federal Institute for Statistics, the estimated population of Tuzla Canton on June 30, 2023, was 430,571, comprising 210,259 men and 220,312 women [17]. Medical records of 483 patients evaluated by the Council for Benign and Malignant Thyroid Diseases at the University Clinical Center Tuzla between January 1, 2020, and December 31, 2024, were retrospectively reviewed. The University Clinical Center Tuzla is the only tertiary referral center for confirmed TC in the Tuzla Canton. Patients diagnosed with TC in other health institutions in the canton are referred to their multidisciplinary thyroid council, which allows institutional documentation to record the majority of confirmed cases during the study period. Data were retrospectively extracted from the medical records of patients who were presented at the Council for Benign and Malignant Thyroid Diseases at the University Clinical Center Tuzla by the authors. Extracted variables included sex, age at diagnosis, date and year of histopathological diagnosis, histological type, and mortality data. Each patient was identified using a unique medical record identifier to prevent double inclusion. Prior to analysis, the documents were reviewed for completeness and consistency. Patients who lacked information necessary to conduct the study were excluded. Mortality status and cause of death were determined from available hospital medical records and follow-up documentation. Age-specific incidence rates were calculated for the following age groups: 15-24, 25-34, 35-44, 45-54, 55-64, 65-74, and ≥75 years.

Study population

Patients were eligible for inclusion if they had histopathologically confirmed TC, were aged ≥15 years, and were residents of Tuzla Canton. Patients were excluded if they resided outside Tuzla Canton or had other thyroid malignancies, including lymphoma, sarcoma, mesenchymal tumors, or metastatic tumors involving the thyroid gland. Of the 483 patients reviewed, 142 met the inclusion criteria and were included in the final analysis.

Histopathological classification

Histopathological diagnoses were obtained from the original pathology reports. For the purposes of this study, TCs were grouped into four major histological categories, papillary, follicular, medullary, and anaplastic TC, in accordance with the World Health Organization classification of thyroid tumors. As the study included cases diagnosed between 2020 and 2024, the original diagnoses reflected the WHO classification applicable at the time of diagnosis. ICD-O morphology codes were not available in the retrospective dataset and were therefore not used in the present analysis.

Statistical analysis

All statistical analyses and graphical presentations were performed using R software (R Foundation for Statistical Computing, Vienna, Austria). The year of histopathological diagnosis was used to assign incident cases. Population denominators were obtained from the Federal Institute for Statistics of the Federation of Bosnia and Herzegovina. Because annual population estimates were not available for each study year, the most recent available population estimate for Tuzla Canton from June 30, 2023, comprising 430,571 inhabitants, including 210,259 men and 220,312 women, was used as the denominator for all incidence and prevalence calculations.

Crude annual incidence rates were calculated as the number of newly diagnosed TC cases in each calendar year divided by the estimated population of Tuzla Canton and multiplied by 100,000. Sex-specific incidence rates were calculated using the corresponding male and female population estimates. The average annual incidence rate for the five-year period was calculated by dividing the total number of incident cases by five times the estimated population and multiplying the result by 100,000.

Age-specific incidence rates were calculated for predefined age groups. Age-standardized incidence rates were estimated using the direct standardization method and the European standard population described by Doll and Cook [18]. Ninety-five percent confidence intervals (CIs) for incidence and prevalence rates were estimated assuming a Poisson distribution of cases. Changes in annual incidence between 2020 and 2024 were assessed using simple linear regression, with calendar year as the independent variable and annual incidence rate as the dependent variable. The regression coefficient, coefficient of determination, and p-value were used to describe the direction and statistical significance of the trend.

Five-year period prevalence was defined as the total number of TC cases identified during the study period divided by the estimated population of Tuzla Canton and expressed per 100,000 population.

## Results

In a five-year period, a total of 142 cases of TC were registered, including 108 (76%) female and 34 (24%) male, with a female-to-male ratio of 3.18:1. The median age of all patients was 56 years (interquartile range (IQR) 24), with an overall range from 16 to 88 years and a mean age of 53.4 years (95% CI: 51-56). Female patients had a median age of 55 years (IQR 23; range 16-84) and a mean age of 53 years (95% CI: 49-56), while male patients were slightly older, with a median age of 59 years (IQR 26; range 24-88) and a mean age of 56 years (95% CI: 51-62) (Table 1).

**Table 1:** Descriptive statistics of age among thyroid cancer patients by sex

Variable	Female	Male	Total
Number of patients, n	108	34	142
Minimum age, years	16	24	16
Maximum age, years	84	88	88
Median age, years	55	59	56
Interquartile range, years	23	26	24
Mean age, years	53.0	56.0	53.4
Lower 95% CI for mean age	49	51	51
Upper 95% CI for mean age	56	62	56

**Figure 1:**
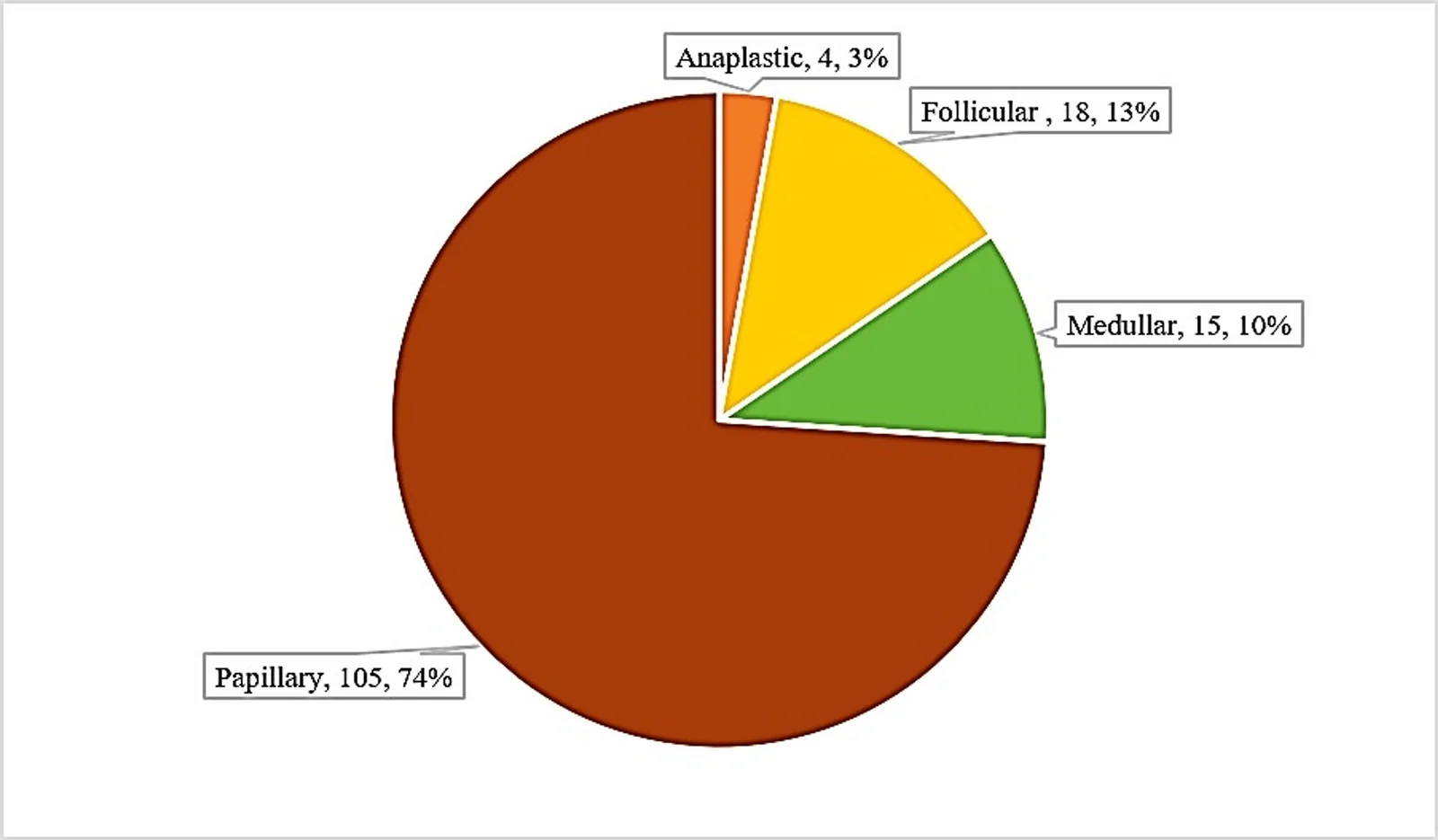
Histological distribution of thyroid cancer cases

Papillary TC was the most common histological type, identified in 105 of 142 patients (74%). Follicular carcinoma was diagnosed in 18 patients (13%), medullary carcinoma in 15 patients (10%), and anaplastic carcinoma in four patients (3%) (Figure 1).

The average annual incidence of TC in the study population over the observed period was 6.59 per 10⁵ inhabitants (95% CI: 5.55-7.77). When stratified by sex, the average annual incidence was 3.23 per 10⁵ in male patients (95% CI: 2.23-4.52) and 9.80 per 10⁵ in female patients (95% CI: 8.04-11.84). After standardization according to the European standard population (Doll & Cook, 1967 [18]), the standardized average annual incidence for the total sample during 2020-2024 was 6.99 per 10⁵ (95% CI: 4.35-9.64). Annual incidence rates of TC, stratified by sex and year from 2020 to 2024, are presented in Table 2.

**Table 2:** Annual incidence rates of thyroid cancer by sex, 2020–2024 Incidence rates are expressed per 100,000 population. Confidence intervals were calculated using the exact Poisson method. CI, confidence interval, n, number of respondents.

	Total		Male		Female	
Years	n	Incidence /10^5^ (95% CI)	n	Incidence /10^5^ (95% CI)	n	Incidence/10^5^ (95% CI)
2020	9	2.74 (1.25 – 5.51)	3	1.87 (0.38 – 5.45)	6	3.56 (1.31 – 7.75)
2021	18	4.14 (2.46 – 6.55)	4	1.87 (0.51 – 4.79)	14	6.25 (3.42 – 10.49)
2022	32	7.4 (5.07 – 10.46)	5	2.37(0.77 – 5.54)	27	12.05 (7.94 – 17.54)
2023	34	7.89 (5.47 – 11.03)	9	4.28 (1.96 – 8.12)	25	11.16 (7.22 – 16.48)
2024	49	10.45 (7.62 – 13.98)	13	6.18 (3.29 - 10.57)	36	14.29 (9.77 – 20.17)

The five-year period prevalence of TC was 32.98 per 100,000 population (95% CI, 27.78-38.87). The corresponding prevalence estimates were 16.17 per 100,000 among male patients (95% CI, 11.19-22.59) and 49.02 per 100,000 among female patients (95% CI, 40.21-59.18).

**Figure 2:**
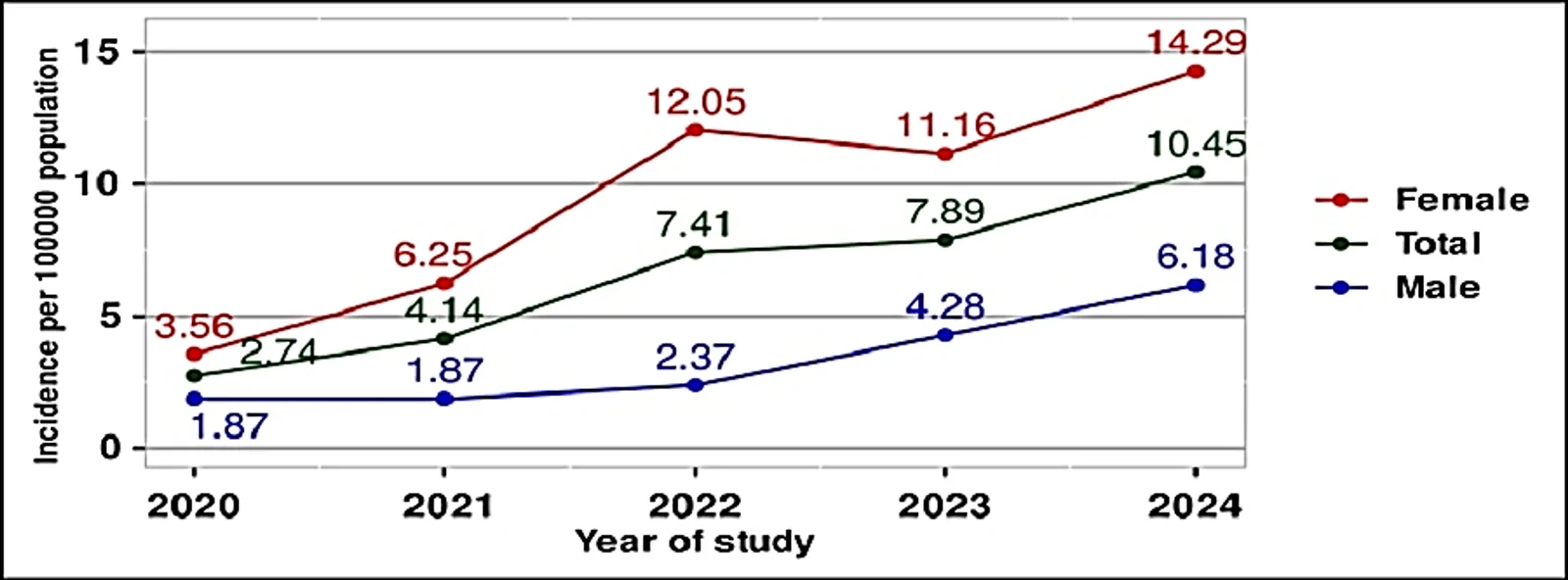
Incidence of thyroid cancer in Tuzla region of Bosnia and Herzegovina during the period 2020–2024 in both gender and complete samples Incidence rates are expressed per 100,000 population

Annual TC incidence rates for male and female patients and the overall population are shown in Figure 2. In the overall population, incidence increased from 2.74 per 100,000 in 2020 to 10.45 per 100,000 in 2024. The highest sex-specific incidence was observed among female patients in 2024, at 14.29 per 100,000 population. Among male patients, incidence ranged from 1.87 per 100,000 in 2020 and 2021 to 6.18 per 100,000 in 2024.

**Figure 3:**
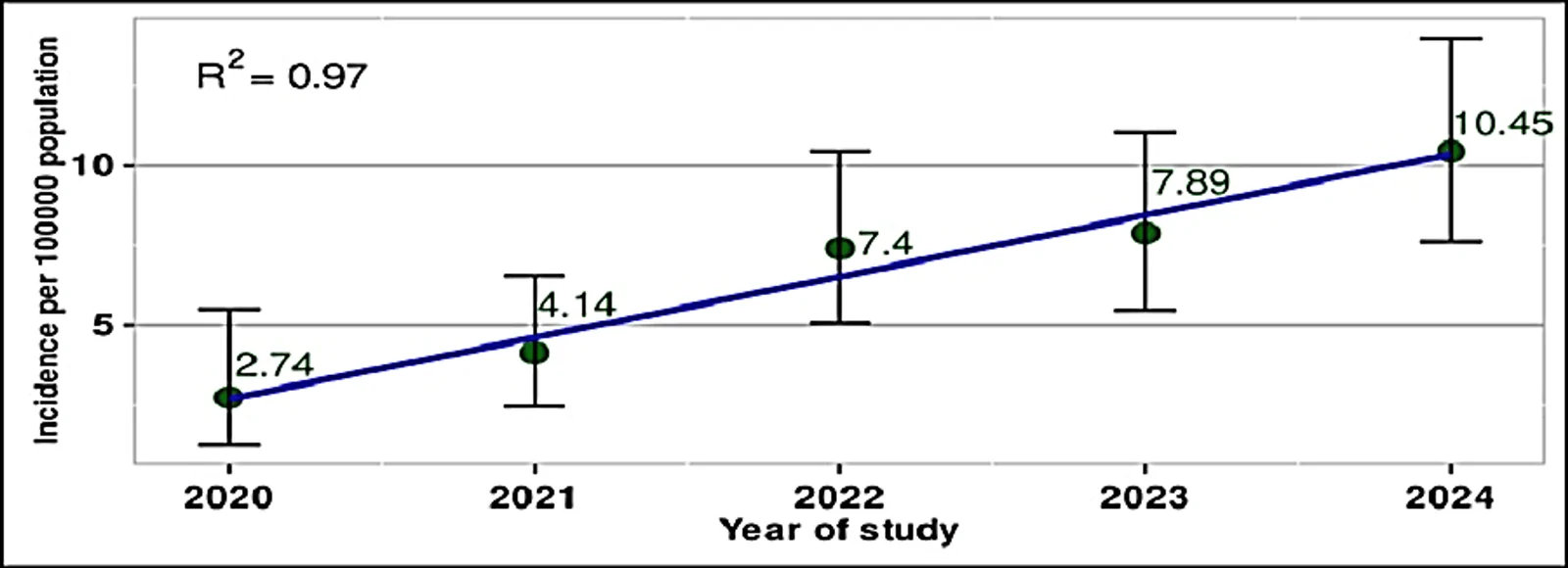
Trend of annual incidence of thyroid cancer in Tuzla region of Bosnia and Herzegovina during the period 2020-2024 Incidence rates are expressed per 100,000 population. The fitted line represents simple linear regression of annual incidence rate by calendar year. R² = 0.966; F(1,3) = 84.80; p = 0.0027

The trend in annual TC incidence during the study period is shown in Figure 3. Overall incidence increased from 2.74 per 100,000 population in 2020 to 10.45 per 100,000 population in 2024. Simple linear regression showed a significant positive annual trend in overall incidence rates (slope = 1.92 per 100,000 population per year, R² = 0.966, F(1,3) = 84.80, p = 0.0027).

**Figure 4:**
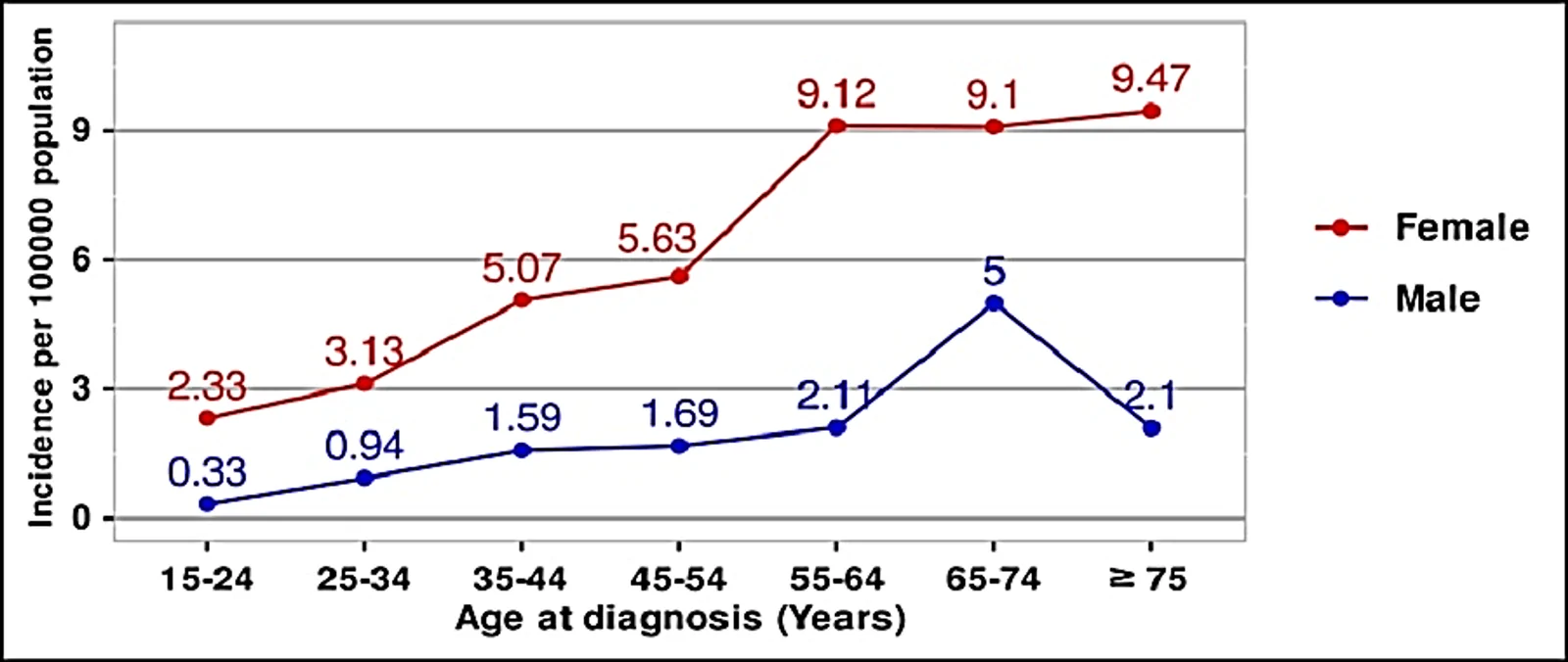
Incidence of thyroid cancer according to age at the time of diagnosis and gender of the patients Incidence rates are expressed per 100,000 population

Age- and sex-specific incidence rates are shown in Figure 4. The highest incidence among female patients was observed in those aged ≥75 years, at 9.47 per 100,000 population. Among male patients, the highest incidence occurred in the 65-74-year age group, at 5.00 per 100,000 population.

During the study period, three of 142 patients (2.1%) died. Two deaths (1.4%) were directly attributable to TC, and both occurred in female patients with anaplastic TC. No TC-related deaths were recorded among patients with other histological types.

## Discussion

This study provides a contemporary overview of TC epidemiology in Tuzla Canton and, to our knowledge, represents one of the most comprehensive regional analyses from Bosnia and Herzegovina. According to the available literature, TC is diagnosed in approximately 5-10% of all thyroid nodules [19-21].

In Bosnia and Herzegovina, based on WHO and GLOBOCAN data for 2022, TC ranked 19th among malignancies by annual incidence, while lung cancer remained the most common malignancy. A total of 20 TC-related deaths were reported in the country [21]. Significant differences in TC incidence between countries are likely due to variations in diagnostic protocols, urban versus rural populations, and differing exposure to etiological factors such as radiation and mutagens [19-21].

During the five-year period from 2020 to 2024, 142 patients with histopathologically confirmed TC were identified. Women accounted for 76% (n=108) of cases, with a female-to-male ratio of 3.18:1, which is consistent with the well-established female predominance reported in previous studies [22]. According to GLOBOCAN 2020, the prevalence of TC in European countries averages 40-60/100,000 in women and 15-25/100,000 in men, which is very close to the values in our sample [23].

Compared with the previously published study from Tuzla Canton covering the period 1999-2008, the present findings suggest a higher incidence in the more recent period, from 2.74-10.45 per 100,000 during 2020-2025, compared with 3.09-2.67 per 100,000 in 1999-2008 [24]. However, comparisons between the two studies should be interpreted cautiously because of possible differences in population denominators, diagnostic practices, histopathological classification, and case ascertainment. Because the study period began in 2020, the observed increase in TC incidence should also be interpreted in the context of the COVID-19 pandemic. Temporary disruptions in diagnostic services, outpatient evaluations, and surgical activity during the early pandemic period may have contributed to lower case detection in 2020, followed by a subsequent rebound as healthcare services resumed.

The marked difference in incidence between women and men is consistent with previous epidemiological reports. Several mechanisms have been proposed to explain this pattern, including hormonal and reproductive factors, greater use of health services among women, and more frequent evaluation of thyroid nodules. Estrogen signaling has also been investigated as a possible biological contributor because estrogen receptors are expressed in differentiated TC cells [25]. Age-specific incidence increased with age in both sexes. The highest incidence among women was observed in those aged ≥75 years, whereas among men the peak occurred in the 65-74-year age group [26]. Regional studies have reported somewhat different age distributions. For example, Slijepčević et al. observed the highest incidence among women aged 50-59 years in Serbia [27]. Our findings are consistent, with the peak incidence among women occurring at 55-64 years. Differences in age-specific patterns may reflect variation in population structure, diagnostic intensity, sample size, and referral practices.

In comparison with Croatia, during 1998-2010, TC incidence in women increased from 6 to 13/100,000, and in men from 1.4 to 3.2/100,000 [28]. Our data demonstrate a similar rising trend. According to these epidemiological data, Croatia ranks fourth in Europe in terms of average TC incidence (11.4/100,000), following Lithuania (15/100,000), Italy (13.5/100,000), and Austria (12.4/100,000). According to the UN Scientific Committee report on the consequences of the Chernobyl nuclear disaster, Croatia was identified as one of the most highly contaminated territories. However, a study by Šimunović et al. (2000), analyzing the periods 1989-1990 and 1998-1999 compared with 1980-1981, did not show a significant difference in the average age of TC patients before and after the disaster, which contrasts with other studies that found TC, particularly among children, 3-4 years post-exposure [29].

Compared with a 2021 study from north Macedonia, prevalence in our cohort was higher in both women (49.02 vs. 32.61 per 100,000) and men (16.17 vs. 9.27 per 100,000) [30]. This may reflect increased detection of papillary carcinoma and improved survival.

The percentage distribution of histological types in the study by Vučemilo et al. shows papillary carcinoma ranging from 81 to 87%, follicular carcinoma from 12.8 to 8.1%, medullary carcinoma from 4.5 to 4.1%, and anaplastic carcinoma from 1.7 to 0.9% [31]. These findings closely align with the distribution observed in our study, further supporting the consistency of histopathological patterns of TC across comparable populations.

Compared with the only published data for the Tuzla Canton from 2012, covering the period 1999-2008, there was a significant decrease in the frequency of anaplastic and follicular carcinomas, with a marked increase in the occurrence of papillary and medullary carcinomas [24]. A similar trend has been observed in other studies and may be associated with the introduction of mandatory salt iodization legislation in 1999.

The increasing incidence observed in this study was not accompanied by a substantial number of TC-related deaths. This pattern is compatible with the broader epidemiological observation that increased detection, particularly of papillary carcinoma, may contribute to rising incidence without a parallel increase in mortality. However, the present study did not include tumor size, stage, incidental diagnosis, or diagnostic pathway, and therefore cannot directly determine the contribution of overdiagnosis. The observed increase in incidence may partly reflect improved diagnostic access and greater detection of papillary TC rather than a true increase in disease occurrence alone.

The main strengths of this study are the histopathological confirmation of all included cases and the inclusion of patients from a defined geographic area. The limitations include the retrospective design, the use of a single population estimate as the denominator for the entire study period, the relatively small number of cases, the lack of tumor stage and risk-factor data, and the absence of a population-based cancer registry. Due to the relatively small number of cases in each age group examined in the study, the precision of age-standardized incidence estimates may have been limited. In addition, the study was based on patients evaluated at a single tertiary referral center, and incomplete case capture cannot be excluded. The findings may not be fully generalizable to other regions of Bosnia and Herzegovina because the study was conducted at a single tertiary center and included only residents of Tuzla Canton. Data on potential risk factors and determinants of case detection, including genetic and hereditary predisposition, family history of TC in first-degree relatives, iodine nutritional status, environmental or medical radiation exposure, obesity, autoimmune thyroid disease, access to healthcare, socioeconomic status, and urban-rural residence, were not available and could not be included in the analysis. Therefore, their possible influence on TC occurrence and detection could not be assessed. The term “five-year period prevalence” should be interpreted cautiously because the analysis included cases identified during the study period rather than all individuals living with previously diagnosed TC in the population. Longer-term, population-based studies and the establishment of a national cancer registry are needed to provide more reliable estimates of TC incidence, prevalence, mortality, and temporal trends in Bosnia and Herzegovina.

## Conclusions

This study provides important regional epidemiological data from Bosnia and Herzegovina, where population-based information on TC remains limited. TC in Tuzla Canton showed a clear female predominance, with papillary carcinoma as the most common histological type. The recorded incidence of TC in Tuzla Canton was lower than estimates reported for many global and European populations, while an upward pattern was observed during the five-year study period. Further long-term, population-based studies and the establishment of a national cancer registry are needed to enable more accurate monitoring of TC incidence, prevalence, mortality, and temporal trends in Bosnia and Herzegovina.
